# Exaggerated blood pressure response to dynamic exercise despite chronic refractory hypotension: results of a human case study

**DOI:** 10.1186/s12882-015-0076-7

**Published:** 2015-06-09

**Authors:** Alice Rogan, Gordon McGregor, Charles Weston, Nithya Krishnan, Robert Higgins, Daniel Zehnder, Stephen M.S. Ting

**Affiliations:** Departments of Renal Medicine and Transplantation, University Hospital Coventry and Warwickshire NHS Trust, Clifford Bridge Road, Coventry, CV2 2DX UK; Departments of Cardiac Exercise Physiology, University Hospital Coventry and Warwickshire NHS Trust, Coventry, UK; Department of Nephrology, Dorset County Hospital NHS Foundation Trust, Dorchester, UK; Division of Metabolic and Vascular Health, The University of Warwick, Coventry, UK

**Keywords:** Blood pressure, Exercise, Chronic hypotension, Haemodialysis

## Abstract

**Background:**

Chronic refractory hypotension is a rare but significant mortality risk in renal failure patients. Such aberrant physiology usually deems patient unfit for renal transplant surgery. Exercise stimulates the mechano-chemoreceptors in the skeletal muscle thereby modulating the sympathetic effects on blood pressure regulation. The haemodynamic response to dynamic exercise in such patients has not been previously investigated. We present a case with severe chronic hypotension who underwent exercise testing before and after renal transplantation, with marked differences in blood pressure response to exercise.

**Case Presentation:**

A 40-year old haemodialysis-dependent patient with a 2 year history of refractory hypotension (≤80/50 mmHg) was referred for living donor renal transplantation at our tertiary centre. Each dialysis session was often less than 2 h and 30 min due to symptomatic hypotension. As part of the cardiovascular assessment, she underwent haemodynamic evaluation with cardiopulmonary exercise testing. Blood pressure normalized during unloaded pedalling but was exaggerated at maximal workload whereby it rose from 82/50 mmHg to a peak of 201/120 mmHg. Transthoracic echocardiography, tonometric measure of central vascular compliance and myocardial perfusion scan were normal. She subsequently underwent an antibody-incompatible renal transplantation and was vasopressor reliant for 14 days during the post-operative period. Eight weeks following transplant, resting blood pressure was normal and a physiological exercise-haemodynamic response was observed during a repeat cardiopulmonary exercise testing.

**Conclusion:**

This case highlights the potential therapeutic role of unloaded leg cycling exercise during dialysis session to correct chronic hypotension, allowing patients to have greater tolerance to fluid shift. It also adds to existing evidence that sympathetic dysfunction is reversible with renal transplant. Furthermore chronic hypotension with preserved exercise-haemodynamic response and cardiovascular reserve should not preclude these patients from renal transplant surgery.

## Background

Chronic refractory hypotension (inter-dialytic systolic blood pressure, SBP <100 mmHg) in long-term dialysis patients is a rare but serious problem. Sustained hypotension between dialysis sessions occurs in 5–10 % and is associated with poor outcomes, including increased mortality [1]. These patients are often deemed unfit for surgery including renal transplant.

Proposed mechanisms focus on inadequate cardiovascular response because of autonomic dysfunction and reduced vascular response to vasopressors [2, 3]. These patients have high levels of circulating noradrenaline but reduced alpha-adrenoreceptor density, which is associated with a blunted pressor response [2]. Reduced sympathetic nerve discharge due to the loss of renal afferent nerve endings is a major causative factor for persistent hypotension in anephric patients [4]. Post-dialysis reduced plasma concentrations of chromogranin A (a protein co-released with cathecholamines) are seen compared to normotensive patients, consistent with blunted sympathetic response [5]. Interleukin-6 and C-reactive protein (CRP) levels have been shown to correlate with mean arterial pressure in hypotensive patients during haemodialysis suggesting an inflammatory role [6].

Current treatment strategies rely on intravascular volume monitoring, alterations in dialysate composition and vasoconstricting drugs [3, 7]. However, there is no definitive management presently. Midodrine and vasopressin therapy have been shown to be effective only in some patients [7]. The most promising technologies are those capable of real-time monitoring of dialysis composition, ultrafiltration rate and temperature and adjust accordingly minute by minute [3]. The levels of sodium, potassium, calcium, bicarbonate and magnesium have all been shown to alter the risk of hypotension during dialysis [7]. Sodium levels appear to have the most significant effect; dialysates with higher sodium concentrations may reduce rates of hypotension during dialysis [8]. Ultrafiltration is associated with a rise in core body temperature secondary to complex autonomic mechanisms; the central response to this is vasodilation. Thus, cooling dialysate from 37 °C to 35° has been shown to maintain intra-dialytic blood pressure (BP) in a number of patients [9].

Although the proposed mechanisms above highlight the hyporesponsiveness of the efferent or afferent sympathetic receptors in causing refractory hypotension, it remains uncertain if stimulation of the skeletal chemosensitive or mechano-receptors through dynamic exercise could modulate haemodynamics in these patients. We present a case of severe chronic hypotension who underwent dynamic exercise testing before and after renal transplantation, with marked differences in blood pressure response to exercise.

## Case report

A 40-year old female haemodialysis-dependent patient had a 2 year history of chronic refractory severe hypotension (≤80/50 mmHg). Previous treatment with α1-adrenergic agonist, mineralocorticoid analog and optimization of dialysate composition with sodium concentration >140 mmol/L and use of bicarbonate buffers had been non-effective. Aetiology of renal failure was reflux nephropathy. She had right native nephrectomy and left native pyeloplasty performed 21 years previously. She received a cadaveric renal graft 13 years ago which failed 9 years later because of chronic allograft nephropathy. The failed graft was removed 3 years previously due to recurrent sepsis. She had been anuric for the last 4 years. There was no history of diabetes, heart failure or ischemic heart disease. She had been on hemodialysis for 6 years following failed peritoneal dialysis. Dialysis adequacy during the 6 months prior to transplant was recorded with urea reduction rate of 75 % and Kt/V (measure of clearance per dialysis factored for patient size) of 1.6.

### Evaluation prior to renal transplant and 8 weeks following transplant

**Fig. 1:**
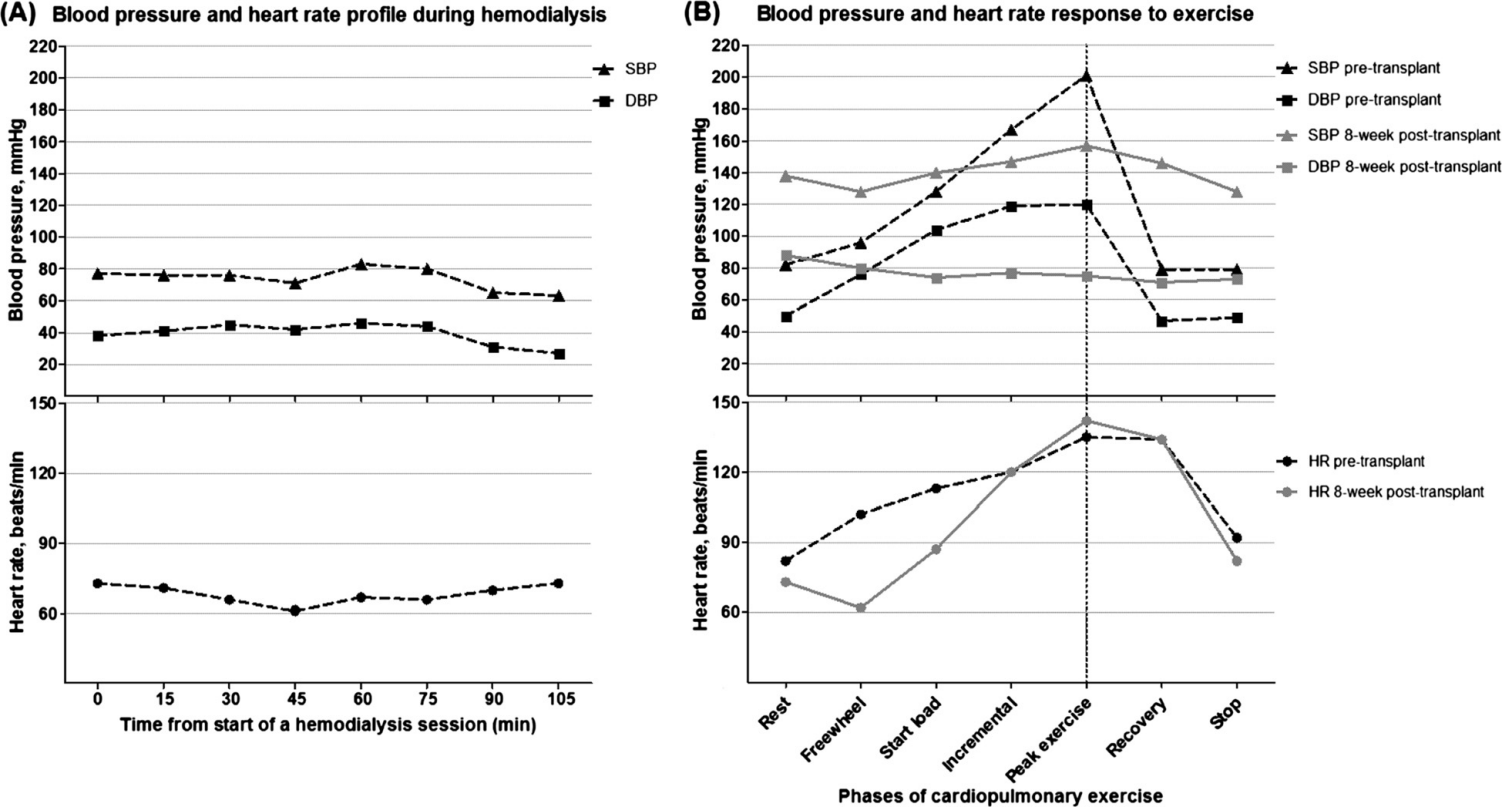
Haemodynamic profile (**a**) during a typical haemodialysis session lasting ≤ 2 h and (**b**) with cardiopulmonary exercise before renal transplant (dotted line) and 8 weeks following transplant (grey line). SBP, systolic blood pressure; DBP, diastolic blood pressure; HR, heart rate

Computed tomography scan revealed a grossly atrophic native left kidney. Transthoracic echocardiography showed normal valves, pericardium and ejection fraction was 62 %. Measures of central vascular stiffness were normal with carotid-femoral pulse wave velocity (PWV) of 5.9 m/s (reference range: 4.5–9.6 m/s) and augmentation index corrected to heart rate (AIx_75_) of 21 % (reference range: 19–24 %). Myocardial perfusion scan was normal. On maximal cardiopulmonary exercise testing (CPET) [10], oxygen consumption at peak exercise (VO_2_peak) was 18 ml/min/kg (73 % of predicted VO_2_peak) (Table 1). Following 3 min rest, pedaling without load (freewheel stage of exercise) produced a 50 % increment in BP from baseline. An exaggerated rise in SBP and diastolic BP (DBP) was recorded at maximal work load, whereby BP rose from 82/50 mmHg to a peak of 201/120 mmHg (ΔSBP/ΔDBP = 119/70 mmHg). At this point, the patient abruptly ceased pedaling and a rapid decline in BP to baseline level was documented (Fig. 1).

**Table 1:** Characteristics before and after kidney transplant

Variables	Baseline	8 week post-transplant	Reference
Clinical			
Body mass index, kg/m^2^	25.6	29.1	-
Resting SBP, mm Hg	82	124	-
Resting DBP, mm Hg	50	82	-
Echocardiography			
LV ejection fraction, %	62.3	63.3	≥50
E/mean e'	8.0	9.9	≤10
Vascular			
Aortic PWV (m/s)	5.9	6.4	4.5–9.6
AIx_75_ (%)	21	28	19–24
Biochemical			
Creatinine, μmol/l	446	139	50–90
eGFR, ml/min/1.73 m^3^	-	39	>60
Hemoglobin, g/dl	13.2	9.1	12–15
Albumin, g/l	44	44	35–50
Cortisol, nmol/l	433	356	150–720
Aldosterone, pmol/l			
Pre-exercise	515	372	28–445
Post-exercise	1342	-	28–445
Renin, mU/l			
Pre-exercise	<9.0	9.0	9.8–23.8
Post-exercise	<9.0	-	9.8–23.8
Cardiopulmonary exercise test			
FEV_1_/FVC, %	78	77	75–80
VO_2_peak, % predicted	73	73	-
VO_2_AT, % of VO_2_peak predicted	41	48	-
Oxygen pulse, ml O_2_/min	9.4	9.3	
Maximal work load, Watt	88	99	-
Endurance time, min	10.4	11.3	-
RER at peak exercise	1.4	1.3	-
HR at rest, beat/min	82	73	-
HR at peak exercise, beat/min	141	142	-

*LV* left ventricular, *E/mean e'* ratio of early transmitral flow velocity to annular mitral velocity (averaged of septal and lateral), *PWV* pulse wave velocity, *AIx*_*75*_ augmentation index corrected to heart rate, *FEV*_*1*_ */FVC*, ratio of forced expiratory volume in 1 s to full vital capacity, *VO*_*2*_ *peak* oxygen consumption at peak exercise, *VO*_*2*_ *AT* oxygen consumption at the point of anaerobic threshold, *RER* respiratory exchange ratio of CO_2_ production to O_2_ consumption, *HR* heart rate

The patient proceeded to have an HLA antibody-incompatible kidney transplant. Immunosuppression consisted of mycophenolate mofetil, tacrolimus and prednisolone. A single dose of 500 mg methylprednisolone intra-operatively and basiliximab 20 mg were given at days 0 and 4. Pre-operatively she underwent seven sessions of HLA antibody removal therapy [11, 12] with cryofiltration, with a further two sessions (day 1 and 2) following surgery and one session of double filtration plasmapheresis on day 3. During the intraoperative period, her BP was supported by infusion of metaraminol. Post-operatively, she was vasopressor reliant for 14 days at which point the creatinine was 133 μmol/l (eGFR 41 ml/min/1.73 m^2^).

Eight weeks following transplantation, her resting BP without anti-hypertensive agent was 124/82 mmHg. Graft function was moderate with eGFR 39 ml/min/1.73 m^2^. On repeat CPET, SBP rose uniformly but DBP did not rise from baseline (ΔSBP/ΔDBP = 19/-13 mmHg) (Fig. 1). VO_2_peak was comparable to the pre-transplant measure but there was improvement in the anaerobic threshold following transplant (VO_2_AT, 48 vs. 41 % of predicted VO_2_peak). Cortisol, renin and aldosterone levels at rest were (pre- vs. post-transplant) 433 vs. 356 nmol/l, <9 vs. 9 mU/l and 515 vs. 372 pmol/l, respectively (Table 1).

## Discussion

To our knowledge this is the first human case study demonstrating an exaggerated BP response to dynamic exercise using CPET in a patient with end-stage renal disease (ESRD) and refractory chronic hypotension. Exercise increases sympathetic and reduces parasympathetic activity resulting in augmented cardiac contractility, stoke volume, heart rate and blood pressure [13]. This adaptive response known as the exercise pressor reflex (EPR) is regulated by mechano- and chemoreceptors in the skeletal muscle [14]. Despite the chronicity of profound hypotension, the patient developed augmentation of BP and tachycardia during the warm-up unloaded phase of exercise. Furthermore, following an abrupt discontinuation of pedaling at maximal stress, a rapid decline in BP was documented. Thus, EPR in the absence of work load appears to be driven by stimulation of the muscle stretch receptors that creates an afferent sympathetic stimulus [14].

In healthy individuals, a regulated increase in cardiac output and a reduction in peripheral vascular resistance (PVR) during exercise produce a rise in SBP while keeping the DBP stable [13], similar to the haemodynamic profile observed at post-transplant in our case. Skeletal muscle PVR is tightly regulated between vasoconstricting and dilating signals to produce a precise functional response from the vascular endothelium allowing maximal muscular work [15]. The exaggerated SBP and DBP at maximal workload but low BP during non-pedaling rest documented prior to transplant could be generated by over-compensatory mechanoreceptor activation in response to hyporesponsive chemoreceptors in the skeletal muscle beds [16]. The latter has been observed in ESRD patients whereby uraemia and inflammatory cytokines are thought to downregulate or cause altered signal transduction in the receptors [1, 17]. Additionally, the absence of renin in our patient prior to transplant suggested not only loss of crucial haemodynamic regulatory renal-derived hormones (eg. renalase and adrenomedullin) [18] but also renal afferent signaling. This along with an inflated sympathetic response from the enhanced activity of the skeletal mechanoreceptors further impedes the ability of skeletal vasodilatation during exercise. When cardiac output is not balanced by increased compliance from peripheral vasculature, the result is sharp rise in BP [13].

ESRD patients are known to have profound exercise intolerance [9] due to abnormal haemodynamic and autonomic responses impairing oxygen delivery to skeletal muscle [14]. Following transplantation, exercise tolerance of the patient improved as demonstrated by the increased endurance time, maximal workload and anaerobic threshold. This suggests restored autonomic function and improved oxygen delivery to muscles due to regulated vasodilatation in exercising skeletal muscle capillary beds. Normalization of BP with transplant was also associated with presence of plasma renin and a reduction in pre-exercise levels of stress hormones (aldosterone and cortisol).

### Limitations

Unfortunately a post-exercise aldosterone level was not obtained due to an analysis problem but we propose this level would have been much lower than that seen before transplant. Therefore, restitution of renal-derived regulatory hormones and renal afferent nerves with a transplant were the likely explanation for the correction of chronic hypotension and EPR. Data on muscle mass or strength were not collected in this study. However, exercise measurements of gases indicated the lack of major skeletal pathology. Despite being hypotensive at rest, this patient was able to complete a maximal exercise testing beyond the point of anaerobic metabolism, achieving an endurance time of 10.4 min (if less than 8 min, muscular deconditioning could play a role). Also, at peak exercise, the objective measure of volitional fatigue determined by the respiratory exchange ratio (RER = ratio of CO_2_ output to O_2_ uptake) was 1.4 whereby a sustained RER of 1.1 is a minimum target level for a maximal test. Although the health related quality of life data was not available, we recognized that the physiological BP response, dialysis independence and increased exercise capacity following transplant could all lead to an improved overall quality of life.

## Conclusion

Our findings suggest there may be a therapeutic role of dynamic exercise in ESRD to correct chronic hypotension, at least temporarily. Unloaded cycling exercise during dialysis sessions may allow these patients to have greater tolerance to fluid shift. Our results add to existing evidence that sympathetic dysfunction is reversible with renal transplant [1]. Furthermore chronic hypotension with preserved exercise-haemodynamic response and cardiovascular reserve should not preclude these patients from transplant surgery.

## Consent

Written informed consent was obtained from the patient who was assessed in a clinical study that was approved by the Black Country Research Ethics Committee (REC:09/H1202/113). A copy of the written consent is available for review by the Editor of this journal.

## Abbreviations

SBP: Systolic blood pressure
DBP: Diastolic blood pressure
PVR: Peripheral vascular resistance
eGFR: Estimated glomerular filtration rate
Kt/V: Measure of clearance per dialysis factored for patient size)
ESRD: End stage renal disease
EPR: Exercise pressor reflex
CPET: Cardiopulmonary exercise testing
VO_2_peak: Peak (maximal) oxygen uptake
VO_2_AT: Oxygen uptake at anaerobic threshold
PWV: Pulse wave velocity
AIx_75_: Augmentation index corrected to heart rate 75 beats/min
